# BMI-Stratified Exploration of the ‘Obesity Paradox’: Heart Failure Perspectives from a Large German Insurance Database

**DOI:** 10.3390/jcm13072086

**Published:** 2024-04-03

**Authors:** Anastasia J. Hobbach, Jannik Feld, Wolfgang A. Linke, Jürgen R. Sindermann, Patrik Dröge, Thomas Ruhnke, Christian Günster, Holger Reinecke

**Affiliations:** 1 Department of Cardiology I, Coronary, Peripheral Vascular Disease and Heart Failure, University Hospital Münster, 48149 Münster, Germany; 2 Institute of Biostatistics and Clinical Research, University of Münster, 48149 Münster, Germany; 3 Institute of Physiology II, University of Münster, 48149 Münster, Germany; 4 AOK Research Institute (WIdO), AOK-Bundesverband, 10178 Berlin, Germanythomas.ruhnke@wido.bv.aok.de (T.R.); christian.guenster@wido.bv.aok.de (C.G.)

**Keywords:** obesity paradox, obesity, chronic heart failure, cardiovascular risk factors, body mass index, long-term outcomes, Germany, real-world data

## Abstract

**Background:** The global rise of obesity and its association with cardiovascular risk factors (CVRF) have highlighted its connection to chronic heart failure (CHF). Paradoxically, obese CHF patients often experience better outcomes, a phenomenon known as the ‘obesity paradox’. This study evaluated the ‘obesity paradox’ within a large cohort in Germany and explored how varying degrees of obesity affect HF outcome. **Methods**: Anonymized health claims data from the largest German insurer (AOK) for the years 2014–2015 were utilized to analyze 88,247 patients hospitalized for myocardial infarction. This analysis encompassed baseline characteristics, comorbidities, interventions, complications, and long-term outcomes, including overall survival, freedom from CHF, and CHF-related rehospitalization. Patients were categorized based on body mass index. **Results**: Obese patients encompassed 21.3% of our cohort (median age 68.69 years); they exhibited a higher prevalence of CVRF (*p* < 0.001) and comorbidities than non-obese patients (median age 70.69 years). Short-term outcomes revealed lower complication rates and mortality (*p* < 0.001) in obese compared to non-obese patients. Kaplan–Meier estimations for long-term analysis illustrated increased incidences of CHF and rehospitalization rates among the obese, yet with lower overall mortality. Multivariable Cox regression analysis indicated that obese individuals faced a higher risk of developing CHF and being rehospitalized due to CHF but demonstrated better overall survival for those classified as having low-level obesity (*p* < 0.001). **Conclusions**: This study underscores favorable short-term outcomes among obese individuals. The ‘obesity paradox’ was confirmed, with more frequent CHF cases and rehospitalizations in the long term, alongside better overall survival for certain degrees of obesity.

## 1. Introduction

The prevalence of obesity has surged globally, marked by a significant increase in Germany where the number of overweight individuals doubled between 1975 and 2016 [1]. Alongside this rise in obesity, the prevalence of associated comorbidities has also escalated, notably impacting risk factors associated with cardiovascular diseases (CVD) such as arterial hypertension (AHT), diabetes mellitus (DM), and dyslipidemia [2,3,4]. These risk factors, in combination with obesity, have an impact on the different types of heart failure (HF). They serve as direct determinants in the pathogenesis of HF with preserved ejection fraction (HFpEF) [5] and triggers for the development of coronary heart disease (CHD) and myocardial infarction (MI) [6]; both are major contributors to HF with reduced ejection fraction (HFrEF). 

The association between obesity and HF has been observed across varying degrees of obesity [7], and across sexes and diverse adiposity measures [8]. However, despite obesity serving as a direct risk factor for HF and exacerbating factors linked to HF, several clinical studies have revealed a surprising, inverse relationship between obesity and HF outcomes [9,10,11,12,13,14,15]. Contrary to anticipated outcomes, a subset of HF-diagnosed individuals presenting with higher body mass index (BMI) or classified as obese demonstrated favorable prognoses and heightened survival rates compared to their normal-weight or under-weight counterparts. This phenomenon, referred to as the ‘obesity paradox’ in HF, has sparked considerable debates and remains incompletely elucidated. Proposed explanations allude to heightened metabolic reserve, neurohormonal alterations, or confounding risk factors like age, sex, and type of HF [16]. Additionally, certain literature indicates a U-shaped relationship between BMI and overall survival in HF, suggesting that the ‘obesity paradox’ weakens in severe obesity and might primarily be applicable to moderately overweight or mildly obese individuals [17,18,19]. However, the majority of existing studies have been limited to small patient cohorts or were predominantly conducted in Asian populations, potentially limiting the generalizability of their findings across diverse ethnicities. Our study seeks to contribute to this ongoing debate by evaluating the ‘obesity paradox’ in HF using extensive real-world data sourced from the health claims data of the largest public German insurance. Our validation of the ‘obesity paradox’ in HF in a cohort of this magnitude bears substantial implications for future HF management approaches. Results may help guide healthcare providers in optimizing treatment strategies, while prompting further investigation into the intricate mechanisms underpinning the ‘obesity paradox’. 

## 2. Materials and Methods

### 2.1. Data Source

Health claims data were obtained anonymized from the *Allgemeine Ortskrankenkasse* (AOK)—Die Gesundheitskasse (local healthcare fund), the largest public health insurance provider in Germany. The AOK comprises a network of eleven regional health insurance companies covering more than 26 million insured individuals [20]. Enrollment in the AOK is unrestricted and available to all residents in Germany, irrespective of geographical location, occupation, income level, age, or health status. We obtained anonymized health claims data from AOK Research Institute (WIdO) for cardiovascular diseases.

### 2.2. Study Population

**Figure 1:**
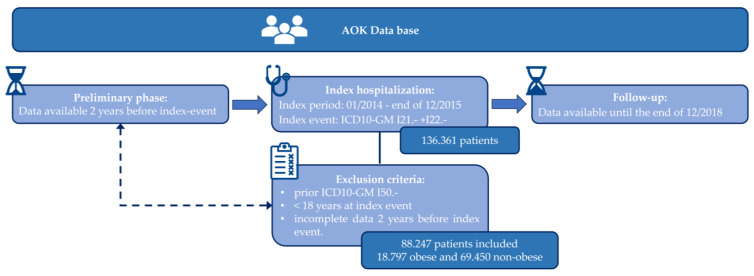
**Composition of the study cohort.** Data utilized in this study were sourced from the comprehensive AOK database. Data collection began two years prior to the index event, with an index period ranging from January 2014 to the end of December 2015. The cohort comprised 88,247 patients, categorized into 18,797 obese and 69,450 non-obese individuals, after excluding those under 18 years old at the index event and those with incomplete data. Follow-up information was available until December 2018. ICD10-GM, International Classification of Diseases, 10th Revision, German Modification.

The dataset encompassed records of 88,247 patients without a prior diagnosis of chronic heart failure (CHF) (ICD-10-GM I50.-) at the index stage and who experienced an index hospitalization with a principal diagnosis of MI (ICD-10-GM I21.-, I22.-) occurring between 1 January 2014, and 31 December 2015. Included within this dataset were comprehensive inpatient and outpatient records spanning two years prior to the index hospitalization, with a subsequent follow-up period extending until 31 December 2018. All individuals aged ≥18 years were included, except those with implausible or missing data (Figure 1). Patients were excluded if their data on gender, date of birth, or date of death were missing or inconsistent. All other variables were defined using ICD, OPS, or ATC Codes. If no relevant code was identified within a predefined timeframe, the variable was assigned a value of zero. Consequently, we established a baseline period of two years preceding the index hospitalization. Patients who were not insured by AOK throughout the entire baseline period (e.g., due to changing insurance providers or extended periods abroad) and thus had incomplete data were also excluded. 

Individuals were categorized into subgroups based on their BMI: obese individuals (BMI ≥ 30 kg/m^2^) and non-obese ones (BMI < 30 kg/m^2^). Among the obese, further subdivisions were made based on the severity of obesity according to ICD10-GM coding (obesity degree I: BMI ≥ 30 to <35 kg/m^2^; obesity degree II: BMI ≥ 35 to <40 kg/m^2^; obesity degree III: BMI ≥ 40; obesity of unknown degree). 

Baseline characteristics for each individual were obtained retrospectively two years prior to the index hospitalization, encompassing cardiovascular risk factors (CVRF) (e.g., AHT, DM, dyslipidemia, nicotine abuses); cardiovascular comorbidities (e.g., previous MI, previous stroke, atrial flutter/fibrillation (AFL/AF), peripheral artery disease (PAD), cerebrovascular disease (CeVD), chronic kidney disease (CKD), ischemic cardiomyopathy (ICM), dilated cardiomyopathy (DCM), hypertrophic obstructive cardiomyopathy (HOCM), hypertrophic cardiomyopathy (HCM), restrictive cardiomyopathy (RCM), arrhythmogenic right ventricular cardiomyopathy (ARVC), iron deficiency, obstructive sleep apnea syndrome (OSAS), left bundle branch block (LBBB), left/right heart failure (L/RHF)); with cardiovascular comorbidities-associated interventions (previous percutaneous coronary intervention (PCI), previous coronary artery bypass grafting (CABG), implanted cardiac device); malignancies; the occurrence and severity grading of HF symptoms (dyspnea, oedema, NYHA classes I–IV).

The pharmacological therapy (including platelet activation inhibition (PAI), oral anticoagulation (OAC), combination therapies of PAI and OAC, angiotensin receptor inhibitors (ACEi), angiotensin receptor blocker (ARB), betablockers, diuretics, ivabradine, mineralocorticoid receptor antagonist (MRA), sodium glucose cotransporter-2 inhibitor (SGLT-2i), and digitalis) if prescribed within a 90-day period preceding the index event was available and analyzed. Additionally, patients were assessed based on the number of distinct drug classes that were prescribed, aligning with the recommended HF therapy at the time of data collection (ACEi/ARB, betablockers, MRA, diuretics, ivabradine). 

Complications during the index hospitalization were defined as acute renal failure, renal replacement therapy, shock, in-hospital resuscitation or the combination of shock, resuscitation and left ventricular (LV) support. Outcomes encompassed death within the case chain and overall mortality. 

Primary endpoints included overall survival, freedom from rehospitalization due to CHF, and freedom from CHF. 

The International Classification of Diseases, 10th Revision, German Modification (ICD-10-GM) was utilized for analyses concerning diagnoses (Table S1). The anatomical therapeutic chemical classification system (ATC) was employed for the analysis of prescribed pharmaceuticals and *Operationen- und Prozedurenschlüssel* (OPS) codes for encoding medical procedures, surgeries, and treatments (Tables S2 and S3).

### 2.3. Ethical Approval 

The Institutional Review Board was duly informed and provided unequivocal approval for the retrospective utilization of anonymized datasets procured from the AOK’s AOK Research Institute (Wissenschaftliches Institut der AOK, WIdO) (file reference: 2019-212-f-S; Ethics Committee of Muenster, Germany).

### 2.4. Statistical Analyses

We used multivariable Cox regression models to analyze the endpoints of overall survival, freedom of CHF, and freedom of rehospitalization due to CHF. The models for freedom of CHF and freedom of rehospitalization due to CHF considered death as a competing risk. For this purpose, we used Fine and Gray’s method to estimate sub-distributional hazard ratios (HRs) in these models. The models included risk profiles of patients at baseline. To assess the impact of severity of HF on the obesity paradox, we repeated the multivariable Cox regression models for the endpoints overall survival and freedom of rehospitalization due to CHF separated by NYHA classification. The hazard ratios of different obesity stages are given in the Supplemental Materials (Tables S4 and S5).

All presented confidence intervals (CI) are standard and unadjusted, and the *p*-values are two-sided, purely descriptive, and unadjusted, and are interpreted accordingly. *p*-values < 0.05 were considered statistically noticeable. Furthermore, we performed univariate analyses for the endpoints of overall survival, freedom from CHF, and freedom from rehospitalization due to CHF to obtain event rates at selected time points. Freedom of CHF and freedom of rehospitalization due to CHF were analyzed with competing risk models by calculating the cumulative incidence, where death was considered as a competing risk. Overall survival was analyzed with the Kaplan–Meier estimator. We did this time-to-event analysis stratified by obesity status. Moreover, medication rates 90 days after the index hospitalization were estimated using competing risk models, considering death as a competing risk. Statistical analyses were performed using R version 4.0.2 (22 June 2020), R Foundation, Vienna, Austria.

## 3. Results

We assessed a total of 88,247 patients without a prior diagnosis of CHF who experienced an index hospitalization due to acute MI during the specified index period. Baseline characteristics were analyzed retrospectively for a period of two years preceding the index hospitalization, followed by a subsequent observational follow-up period extending until 31 December 2018. The median follow-up duration was 3.99 years, with an interquartile range (IQR) of 1.04 years. We further assessed the rates of outcome and complications from the index hospitalization up to 90 days following discharge. Additionally, multivariable Cox regression analyses were performed to evaluate the long-term outcomes, defined as overall survival, freedom from CHF, and freedom from rehospitalization due to CHF (Figure 1). 

### 3.1. Baseline

Within the entire cohort, 21.3% of individuals were diagnosed with obesity, with 40.66% of them being female (Table 1). The median age for individuals with obesity was 68.69 years (IQR 18.94 years), while non-obese individuals were older with a median age of 70.69 years (IQR 21.40 years). Comorbidities of interest, identified two years prior to the index hospitalization, were more prevalent in obese individuals compared to non-obese counterparts (Table 1). Specifically, obesity was associated with higher occurrence rates of AHT, DM, dyslipidemia, previous MI, previous stroke, AFL/AF, PAD, CeVD, CKD, and OSAS (Table 1). Baseline findings revealed that obese individuals had more frequently prior interventions related to CVD (prior PCI or CABG) compared to non-obese ones (11.96% vs. 7.27%, *p* < 0.001). Some patients had already been stratified into NYHA classes at baseline, allocating 2.9% to class I, 7.8% to class II, 11.40% to class III, and 13.18% to class IV, indicating a relatively symptomatic cohort (Table 1). While no statistically noticeable distinctions were observed between obese and non-obese patients in their distribution among NYHA classes, specific symptoms such as edema and dyspnea were more prevalent in the obese subgroup. Nicotine abuse was statistically more common among obese compared to non-obese individuals (26.48% vs. 24.51%, *p* < 0.001). Further, prescription rates for medical therapies were notably higher among obese patients across all considered drug categories analyzed in this study compared to their non-obese counterparts (Table 1). 

### 3.2. Outcome and Follow-Up

The incidences of the severe complications (shock (6.94% vs. 8.13%, *p* < 0.001), in-hospital resuscitation (6.63% vs. 7.06%, *p* = 0.041), and the combination of shock, resuscitation, and LV support (11.14% vs. 12.41%, *p* < 0.001)) were more frequent in non-obese individuals than in the obese cohort during the index hospitalization (Table 2). Conversely, complications of renal replacement therapy (3.30% vs. 2.49%, *p* < 0.001) and acute renal failure (6.85% vs. 5.95%, *p* < 0.001) demonstrated higher occurrence rates in obese vs. non-obese individuals during index hospitalization (Table 2). Rates for mortality within the case chain (8.9% vs. 10.55%, *p* < 0.001) and overall death (7.57% vs. 9.26%, *p* < 0.001) were elevated among non-obese patients during the index hospitalization, highlighting the phenomenon of the ‘obesity paradox’ (Table 2). The length of the hospital stay did not distinguish between obese and non-obese patients (both at 7 days, *p* < 0.001). 

### 3.3. Long-Term Outcomes

**Figure 2:**
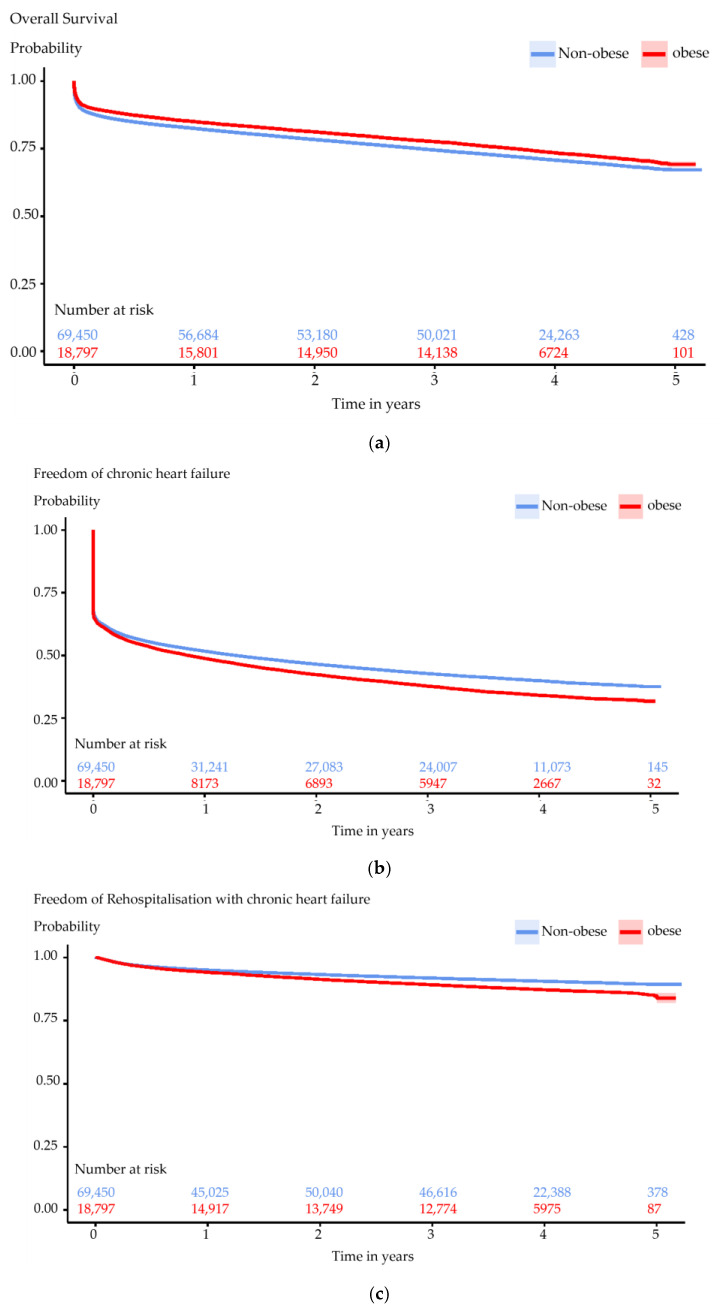
**Primary outcomes.** The estimated overall survival rates (**a**), the estimated cumulative incidence of the primary outcome ‘freedom from chronic heart failure (CHF)’ (**b**), and the estimated cumulative incidence of the primary outcome ‘freedom from rehospitalization due to chronic heart failure (CHF)’ (**c**) stratified between obese (depicted in red) and non-obese (depicted in blue). These estimates were derived using the Kaplan–Meier methodology.

The unstratified Kaplan–Meier estimations revealed the overall survival rate to be 85.0% (95% confidence interval (CI): 84.5%, 85.5%; *p* < 0.001) for obese and 82.5% (95% CI: 82.3%, 82.8%; *p* < 0.001) for non-obese patients one-year post-index hospitalization, exhibiting a decrease with longer follow-ups (81.2% (95% CI: 80.7%, 81.8%; *p* < 0.001) for obese and 75.4% (95% CI: 78.1%, 78.7%; *p* < 0.001) for non-obese patients after two years, 69.2% (95% CI: 68.2%, 70.2%; *p* < 0.001) for obese and 67.2% (95% CI: 66.7%, 67.7%; *p* < 0.001) for non-obese patients after five years) (Figure 2a). Notably, individuals with severe obesity (BMI ≥ 40) initially demonstrated survival rates similar to other obesity groups within the first two years post-event. Thereafter, survival rates for severe obesity declined, approaching levels observed in non-obese individuals after four years (Figure S1a).

In terms of freedom from CHF, the unstratified Kaplan–Meier estimations initially reported rates of 48.7% (95% CI: 48.0%, 49.5%; *p* < 0.001) for obese and 51.6% (95% CI: 51.3%, 52.0%; *p* < 0.001) for non-obese patients one year post-index hospitalization, declining over time: 42.3% (95% CI: 41.6%, 43.0%; *p* < 0.001) for obese and 46.5% (95% CI: 46.1%, 46.9%; *p* < 0.001) for non-obese patients after two years; 31.7% (95% CI: 30.7%, 32.6%; *p* < 0.001) for obese and 37.6% (95% CI: 37.1%, 38.0%; *p* < 0.001) for non-obese patients after five years (Figure 2b). Consistently, obese patients exhibited lower rates of freedom from CHF compared to non-obese individuals throughout the observation period, with notably higher susceptibility observed among those with severe obesity (Figure S1b). 

For freedom from rehospitalization due to CHF, the unstratified Kaplan–Meier estimations indicated rates of 48.8% (95% CI: 48.0%, 49.5%; *p* < 0.001) for obese and 51.7% (95% CI: 51.3%, 52.0%; *p* < 0.001) for non-obese patients one year after the index hospitalization, diminishing over time: 42.3% (95% CI: 41.6%, 43.0%; *p* < 0.001) for obese and 46.5% (95% CI: 46.1%, 46.9%; *p* < 0.001) for non-obese patients after two years; 31.7% (95% CI: 30.7%, 32.6%; *p* < 0.001) for obese and 37.6% (95% CI: 37.1%, 38.0%; *p* < 0.001) for non-obese patients after five years (Figure 2c). Similarly, obese patients demonstrated lower rates of freedom from rehospitalization due to CHF compared to their non-obese counterparts within a five-year span following the index event; individuals with severe obesity were at the highest risk (Figure S1c).

### 3.4. Multivariable Analysis of Overall Survival

**Figure 3:**
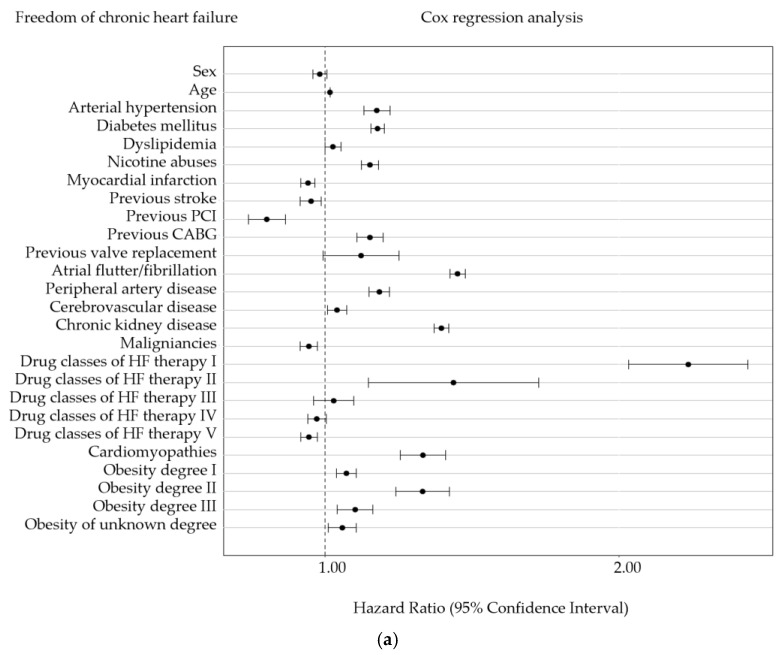

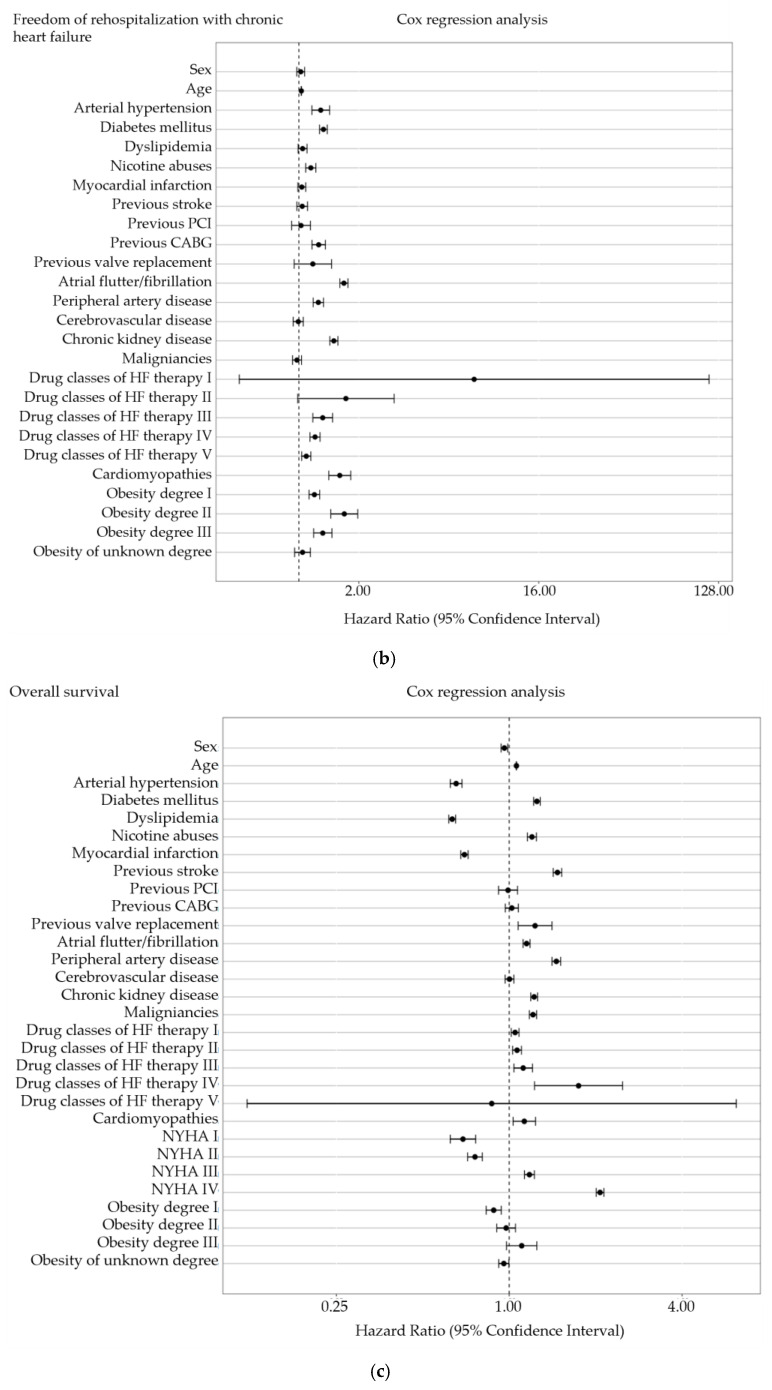
**Multivariable Cox regression analysis was applied for predicting long-term outcomes**. The analysis was conducted for three endpoints: freedom from chronic heart failure (CHF) (**a**), freedom from rehospitalization due to CHF (**b**), and overall survival (**c**). The covariates included in the regression model were age, arterial hypertension, diabetes mellitus, dyslipidemia, nicotine abuses, previous myocardial infarction, previous stroke, previous coronary artery bypass grafting, previous PCI, cerebrovascular disease, chronic kidney disease, atrial flutter/fibrillation, various forms of cardiomyopathy, peripheral artery disease, malignancies, the number of distinct drug classes, and different obesity classes. The models for freedom from CHF and freedom from rehospitalization due to CHF considered death as a competing risk. CABG, coronary artery bypass grafting; HF, heart failure; NYHA, New York Heart Association; PCI, percutaneous coronary intervention.

We also applied a multivariable Cox regression analysis for long-term outcomes, encompassing overall survival, freedom from CHF, and freedom from rehospitalization due to CHF, including age and baseline comorbidities and risk factors (i.e., AHT, DM, dyslipidemia, nicotine abuse, previous PCI, previous CABG, previous MI, previous stroke, AFL/AF, PAD, CeVD, CKD, malignancies, medical therapies, CM, and obesity degree) to elucidate their impact on long-term outcomes. The analysis revealed that irrespective of the degree of obesity, obesity was associated with a higher risk of developing CHF and being rehospitalized (except for the group of unknown obesity) due to CHF (Figure 3a,b). Specifically, patients with severe obesity across NYHA classes I–III were identified as being at high risk for CHF-related rehospitalization (Table S4).

Regarding overall survival, the multivariable Cox regression analysis showed no significantly noticeable impact of obesity on overall survival except for obesity degree I, which was significantly noticeable protective (Figure 3c). The subgroup analysis for the different NYHA classes showed similar, although not statistically noticeable results (Table S5).

## 4. Discussion

The global increase in obesity rates and its associated comorbidities impacting CVRF have intensified the focus on HF and its correlation with obesity. Despite obesity being a recognized risk factor for HF, studies have revealed an unexpected ‘obesity paradox’ in HF outcomes, indicating favorable prognoses and heightened survival rates among obese individuals compared to normal or underweight counterparts [9,10,11,14,15]. Addressing the debates surrounding this paradox, our study validates the existence of the ‘obesity paradox’ in HF for obesity of unknown state and obesity degree I using extensive real-world data from a large German cohort. 

Our findings confirm that obese patients are at higher risk for CVD, exhibiting increased occurrence rates of CVRF and cardiovascular comorbidities compared to non-obese individuals. We demonstrate obese patients have a higher frequency of prior interventions related to CVD before the index hospitalization. Therefore, cardiovascular events might occur at a younger age among obese individuals [21,22], as evidenced by our observation that obese individuals were statistically younger at index hospitalization than their non-obese counterparts. Despite this fact, obese patients exhibited a broader baseline usage of cardiac medications compared to non-obese counterparts, potentially attributable to the higher burden of comorbidities and easier titration of medication doses in obese patients [23]. 

Although obesity clearly heightens the risk of CVD, our findings indicate a better short-term prognosis for obese compared to non-obese patients at index hospitalization. Severe complications, except for renal failure, were more prevalent among non-obese individuals during the index hospitalization, with higher rates of mortality within the case chain and overall mortality. This observation underscores the presence of the ‘obesity paradox’ in our cohort not only for the occurrence of the index event but also for the short-term outcomes post MI during the index hospitalization. The underlying mechanisms contributing to the protective effects of obesity post MI remain incompletely understood but likely involve multiple factors. Further investigations are warranted to elucidate the pathomechanisms underlying the ‘obesity paradox’ in acute cardiovascular events. 

The protective effects of obesity were corroborated by unstratified Kaplan–Meier estimations concerning long-term outcomes, including overall survival, freedom from CHF, and freedom from rehospitalization due to CHF. Our investigations revealed that obesity in general was linked to a heightened incidence of CHF, increased CHF-related rehospitalization rates, but intriguingly, to a lower overall mortality. Unstratified for further risk factors, elevated incidence of CHF and higher rates of CHF-related hospitalization were prominent among patients with severe obesity. After adjusting for other risk factors, our multivariable Cox regression analysis for long-term outcomes, encompassing overall survival and the endpoints freedom from CHF and freedom from rehospitalization due to CHF, indicated obesity degree I as beneficial for overall survival but detrimental for freedom from CHF and freedom from rehospitalization. This mirrors the ‘obesity paradox’ for a moderate level of obesity, demonstrating a higher likelihood for HF but a better overall survival. 

Potential explanations for the pathophysiological mechanisms linking obesity to HF involve hemodynamic alterations, neurohormonal activation, and adipose tissue’s endocrine and paracrine effects [24]. Neurohumoral changes, such as heightened sympathetic nervous system activity, increased catecholamines, and increased activity of the renin–angiotensin–aldosterone system (RAAS) contribute to myocardial remodeling and LV hypertrophy, increasing HF risk [24]. Conversely, obesity correlates with lower B-type natriuretic peptide levels [25], indicating a more favorable hemodynamic profile. These individuals may exhibit a blunted response to RAAS, maintaining higher blood pressures that preserve renal function and enabling tolerance to cardioprotective medications [26]. Further, adipose tissue, recognized as an active organ, secretes inflammatory cytokines and adipokines, potentially exacerbating HF pathogenesis [24]. But over the long term, elevated lipoprotein levels in obesity may neutralize circulating inflammatory endotoxins associated with advanced HF and negative outcomes. Understanding the underlying mechanisms for the increased occurrence of HF but better overall survival in obese individuals would require mechanistic studies. Our study emphasizes the need for such investigations. In patients with severe obesity, overall survival rates were lower and appeared to approximate those of individuals with normal weight over the long term. This U-shaped correlation between BMI and mortality has been corroborated by others [17,18,19]. It can be speculated that severely obese CHF patients experience more substantial advantages from weight reduction compared to slightly overweight and moderately obese patients. Further, it could be that the U-shaped relationship between BMI and outcome in CHF results from the inadequacy of BMI to accurately diagnose genuine pathological adiposity, potentially masking a dose–response correlation between obesity and overall mortality. The group categorized as ‘severe obesity’ may in fact encompass individuals with high adiposity, whereas within the group classified as moderately obese and overweight, individuals with a higher BMI due to increased muscle mass or protective fat distribution could be concealed. Our data suggest that weight reduction, particularly targeting severe obesity, should be promoted as a preventive measure against the development of HF. For those already diagnosed with HF, weight reduction, especially among severely obese individuals, appears to have favorable prognostic implications. However, our data do not suffice to recommend weight reduction for individuals with mild obesity, particularly regarding a potentially protective effect on prognosis. It might be speculated that a general recommendation for weight reduction concerning overall survival in HF patients across all weight categories may not be universally applicable. Therefore, it is crucial to gather additional data using individual body compartments, e.g., waist circumference or body fat percentage, before making definitive conclusions regarding the association between obesity and outcomes in HF.

In conclusion, our study presents valuable insights into the enigmatic ‘obesity paradox’ within HF outcomes. To the best of our knowledge, we are the first to present such extensive real-world data from a substantial German cohort, thus providing crucial insights into this paradoxical phenomenon. Despite obese individuals manifesting heightened cardiovascular risk factors and comorbidities, our study delineates their paradoxically favorable short-term outcomes in HF, demonstrating increased survival rates during the index hospitalization. Additionally, we reveal the association between obesity and escalated HF occurrence and rehospitalization rates, along with notably lower overall mortality in the long term among those with unknown obesity status and those classified as having obesity degree I. It is imperative to note that our investigations do not encompass the underlying mechanistic aspects. Rather, they serve as a prelude, emphasizing the critical need for sustained and specialized research endeavors. The pursuit of further mechanistic elucidation is essential for targeted interventions aimed at deciphering and leveraging the potentially advantageous role of obesity in HF management. 

## 5. Limitations

While our study provides valuable insights into the relationship between obesity and HF using data from a specific insurance database in Germany, it is essential to acknowledge several limitations regarding the generalizability of our findings.

Firstly, the study’s reliance on data from a specific healthcare system may limit the applicability of our results to populations outside of Germany. Healthcare delivery systems, access to care, and patient demographics may differ significantly across countries, affecting the external validity of our findings. Variations in healthcare policies, cultural norms, and lifestyle factors may also influence the prevalence and impact of obesity on HF outcomes in different regions. Furthermore, the characteristics of patients included in our study, such as socio-economic status, comorbidities, and treatment patterns, may not be representative of other populations. The predominance of lower socioeconomic populations within the AOK database, along with the exclusion of privately insured patients, could introduce potential biases in the socioeconomic representation within our study cohort.

Additionally, due to the retrospective nature of our study and reliance on ICD codes, there are limitations in analyzing the impact of weight reduction on prognosis or assessing medication dosages. The inability to differentiate between different types of HF using ICD codes restricts our comprehension of the underlying mechanisms, which could affect the interpretation of our findings. While MI served as the index event, it may not encompass all potential causes of HF, and the occurrence of MI does not exclude other causes or allow a differentiation between HFpEF and HFrEF.

Future research endeavors should aim to address these limitations by incorporating multi-center, multinational collaborations and diverse patient cohorts. Additionally, prospective studies focusing on mechanistic elucidation and the impact of weight reduction strategies on HF outcomes are warranted to provide a more comprehensive understanding of the ‘obesity paradox’ across different healthcare systems and populations.

## Figures and Tables

**Table 1:** Patient characteristics at baseline: non-obese vs. obese.

	Non-Obese (BMI < 30 kg/m^2^)	Obese (BMI ≥ 30 kg/m^2^)	Total	*p*-Value
Number of patients—a.n.	69,450	18,797	88,247	
Female sex—a.n. (%)	23,203 (33.41)	7642 (40.66)	30,845 (34.95)	<0.001
Age median—years (IQR)	70.69 (21.40)	68.69 (18.94)	70.22 (20.82)	<0.001
**Cardiovascular risk factors—a.n. (%)**				
Arterial hypertension (AHT)	60,116 (86.56)	18,050 (96.03)	78,166 (88.58)	<0.001
Diabetes mellitus (DM)	23,506 (33.85)	11,523 (61.30)	35,029 (39.69)	<0.001
Dyslipidemia	50,341 (72.49)	15,532 (82.63)	65,873 (74.65)	<0.001
Nicotine abuses	17,019 (24.51)	4977 (26.48)	21,996 (24.93)	<0.001
**Comorbidities and other risk factors—a.n. (%)**				
Previous myocardial infarction (MI)	21,005 (30.24)	7150 (38.04)	28,155 (31.94)	<0.001
Previous stroke	6705 (9.65)	2144 (11.41)	8849 (10.03)	<0.001
Malignancies	12,080 (17.39)	3291 (12.17)	15,371	0.714
Atrial flutter/fibrillation (AFL/AF)	13,599 (19.58)	4137 (22.01)	17,736 (20.1)	<0.001
Peripheral artery disease (PAD)	6867 (9.89)	2287 (12.17)	9154 (10.37)	<0.001
Cerebrovascular disease (CeVD)	8269 (11.91)	2832 (15.07)	11,101 (12.58)	<0.001
Chronic kidney disease (CKD)	17,332 (24.96)	6276 (33.39)	23,608 (26.75)	<0.001
Ischemic cardiomyopthy (ICM)	4880 (7.02)	1298 (6.91)	6178 (7.0)	0.563
Dilated cardiomyopthy (DCM)	796 (1.15)	224 (1.19)	1020 (1.16)	0.604
Hypertrophic obstructive cardiomyopthy (HOCM)	153 (0.22)	42 (0.22)	195 (0.22)	0.935
Hypertrophic cardiomyopthy (HCM)	191 (0.28)	64 (0.34)	255 (0.29)	0.138
Iron deficiency	777 (1.12)	238 (1.27)	1015 (1.15)	0.093
Obstructive sleep apnea syndrome (OSAS)	2271 (3.27)	2105 (11.2)	4376 (4.96)	<0.001
Left bundle branch block (LBBB)	1685 (2.42)	2552 (2.94)	2237 (2.53)	<0.001
Left heart failure (LHF)	23,086 (33.24)	6415 (34.13)	29,501 (33.43)	0.022
Right heart failure (RHF)	4516 (6.5)	1375 (7.31)	5891 (6.68)	<0.001
**Interventions associated with cardiovascular comorbidities—a.n. (%)**				
Implanted cardiac device	2237 (3.22)	672 (3.58)	2909 (3.3)	0.016
Previous Percutaneous coronary intervention (PCI)	1788 (2.57)	885 (4.71)	2673 (3.03)	<0.001
Previous Coronary artery bypass grafting (CABG)	3262 (4.7)	1363 (7.25)	4625 (5.24)	<0.001
Previous valve replacement	313 (0.45)	134 (0.71)	447 (0.51)	<0.001
**Occurrence and severity grading of HF symptoms—a.n. (%)**				
Dyspnea	5702 (8.21)	2513 (13.37)	8215 (9.31)	<0.001
Oedema	3270 (4.71)	1814 (9.65)	5084 (5.76)	<0.001
New York Heart Association (NYHA) I	2052 (2.95)	532 (2.83)	2584 (2.93)	0.369
NYHA II	5431 (7.82)	1491 (7.93)	6922 (7.84)	0.612
NYHA III	7768 (11.19)	2293 (12.2)	10,061 (11.40)	<0.001
NYHA IV	9121 (13.13)	2508 (13.34)	11,629 (13.18)	0.452
**Pharmacological therapy—a.n. (%)**				
Platelet activation inhibition (PAI)	8135 (11.71)	3005 (15.99)	11,140 (12.62)	<0.001
Oral anticoagulation (OAC)	2057 (2.96)	860 (4.58)	2917 (3.31)	<0.001
PAI+OAC	225 (0.32)	105 (0.56)	330 (0.37)	<0.001
Angiotensin receptor inhibitors/angiotensin receptor blocker (ACEi/ARB)	25,411 (36.59)	9912 (52.73)	35,323 (40.03)	<0.001
Statine	11,287 (16.25)	4709 (25.05)	15,996 (18.12)	<0.001
Betablockers	18,488 (26.62)	7102 (37.78)	25,590 (29.0)	<0.001
Diuretics	3007 (4.33)	1456 (7.75)	4463 (5.06)	<0.001
Ivabradine	171 (0.25)	91 (0.48)	262 (0.3)	<0.001
Mineralocorticoid receptor antagonist (MRA)	603 (0.87)	308 (1.64)	911 (1.03)	<0.001
Sodium glucose cotransporter-2 inhibitor (SGLT-2i)	68 (0.1)	101 (0.54)	169 (0.19)	<0.001
Digitalis	803 (1.16)	200 (1.06)	1003 (1.14)	0.29
Drug classes of HF therapy (None)	36,289 (52.25)	6427 (34.19)	42,716 (48.41)	<0.001
Drug classes of HF therapy (One)	11,744 (19.91)	4947 (26.32)	16,691 (18.91)	
Drug classes of HF therapy (two)	20,047 (28.87)	6661 (35.44)	26,708 (30.27)	
Drug classes of HF therapy (three)	1336 (1.92)	735 (3.91)	2071 (2.35)	
Drug classes of HF therapy (four)	33 (0.05)	26 (0.14)	59 (0.07)	

Qualitative data were assessed using a two-sided χ^2^ test, while quantitative data were evaluated employing a two-sided Wilcoxon test.

**Table 2:** Outcomes and follow-up during index hospitalization: non-obese vs. obese.

	Non-Obese (BMI < 30 kg/m^2^)	Obese (BMI ≥ 30 kg/m^2^)	Total	*p*-Value
Number of patients	69,450	18,797	88,247	
Acute renal failure—a.n. (%)	4133 (5.95)	1288 (6.85)	5421 (6.14)	<0.001
Renal replacement therapy—a.n. (%)	1727 (2.49)	620 (3.3)	2347 (2.66)	<0.001
Shock—a.n. (%)	5646 (8.13)	1304 (6.94)	6950 (7.88)	<0.001
In-hospital resuscitation—a.n. (%)	4901 (7.06)	1246 (6.63)	6147 (6.97)	0.041
Shock/resuscitation/LV support—a.n. (%)	8617 (12.41)	2094 (11.14)	10,711 (12.14)	<0.001
Death within case chain—a.n. (%)	7326 (10.55)	1673 (8.9)	8999 (10.2)	<0.001
Overall mortality—a.n. (%)	6430 (9.26)	1422 (7.57)	7852 (8.9)	<0.001
Length of stay median—days (IQR)	7 (6)	7 (6)	7 (6)	<0.001

Qualitative data were assessed using a two-sided χ^2^ test, while quantitative data were evaluated employing a two-sided Wilcoxon test. Event rates at 90 days were analyzed with competing risk models by calculating the cumulative incidence, where death was considered as a competing risk. IQR, interquartile range; LV, left ventricular.

## Data Availability

All data are stored in a central database at the AOK Research Institute (WIdO, Berlin). We received aggregated and anonymized data of all patients meeting the above-mentioned inclusion criteria. The authors confirm that the data utilized in this study cannot be made available in the manuscript, the supplemental files, or in a public repository due to German data protection laws (‘Bundesdatenschutzgesetz’, BDSG). Generally, access to data of statutory health insurance funds for research purposes is possible only under the conditions defined in German Social Law (SGB V § 287). Requests for data access can be sent as a formal proposal specifying the recipient and purpose of the data transfer to the appropriate data protection agency. Access to the data used in this study can only be provided to external parties under the conditions of the cooperation contract of this research project and after written approval by the sickness fund. For assistance in obtaining access to the data, please contact wido@wido.bv.aok.de.

## Supplementary Materials

The following supporting information can be downloaded at: https://www.mdpi.com/article/10.3390/jcm13072086/s1, Figure S1: Primary outcome across obesity degree; Table S1: Used Health Problems 10th Revision (ICD-10 GM) codes; Table S2: Used Anatomical Therapeutic Chemical classification system (ATC) codes.; Table S3: Used German procedure classification system (OPS) codes. Table S4: Hazard ratios of the different obesity stages regarding Cox regression models for freedom from rehospitalization. Table S5: Hazard ratios of the different obesity stages regarding Cox regression models for overall survival.
