# Supplementary material for: BMI-Stratified Exploration of the ‘Obesity Paradox’: Heart Failure Perspectives from a Large German Insurance Database
Source: J Clin Med. 2024 Apr 3;13(7):2086. doi: 10.3390/jcm13072086 (PMC11012389; doi:10.3390/jcm13072086)
Supplement: Supplementary file 1 [file jcm-13-02086-s001.zip › Supplement_FigureS1.pdf]

Supplement: Figure S1: Primary Outcome across obesity degrees.

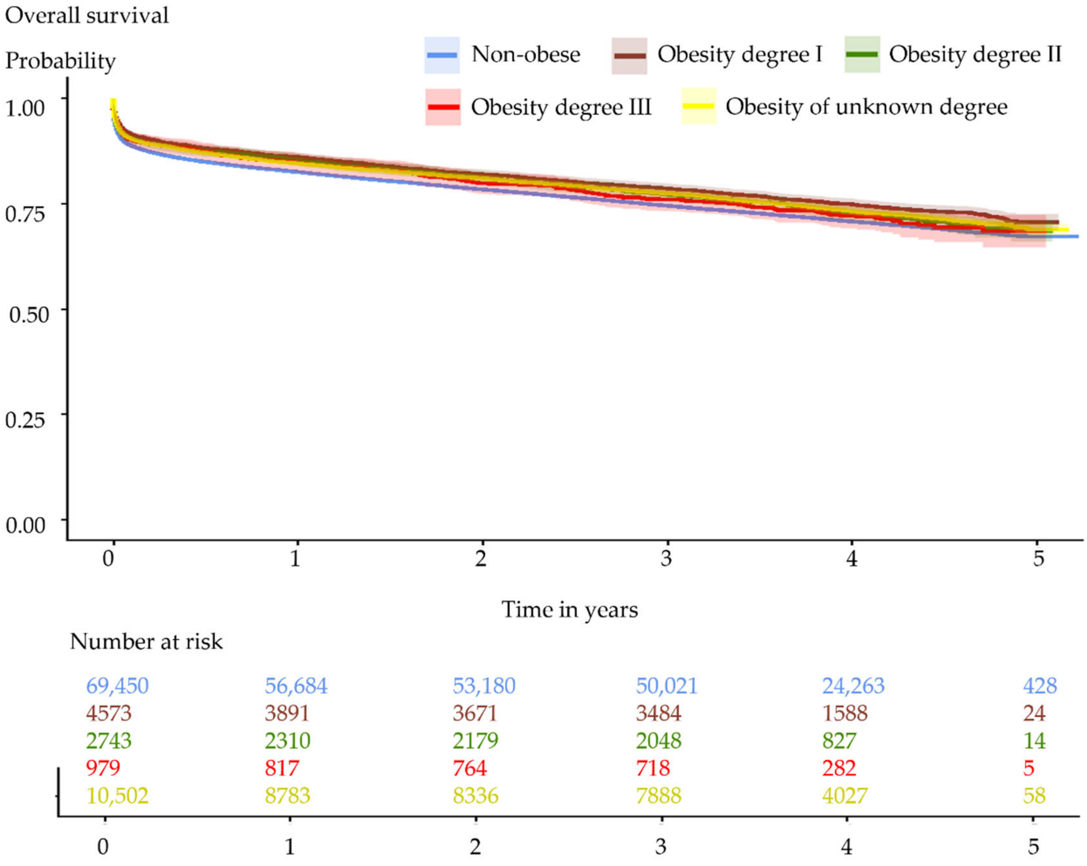

(**a**)

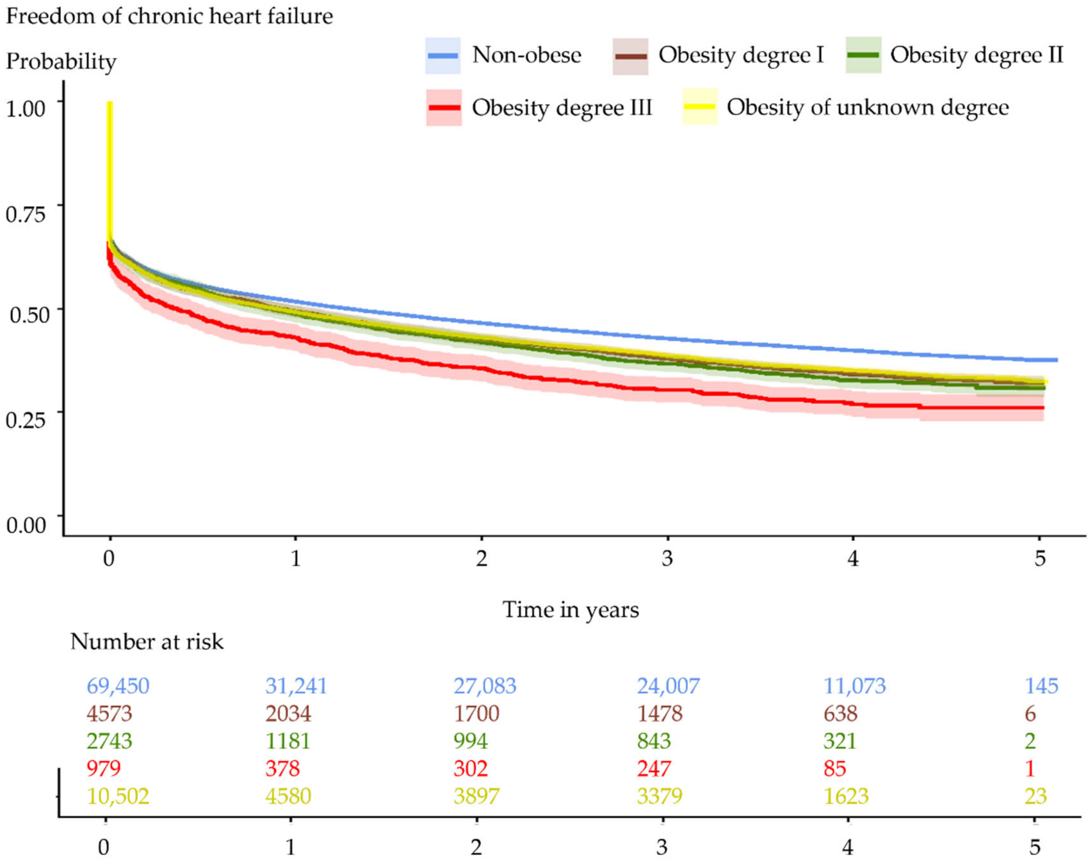

(**b**)

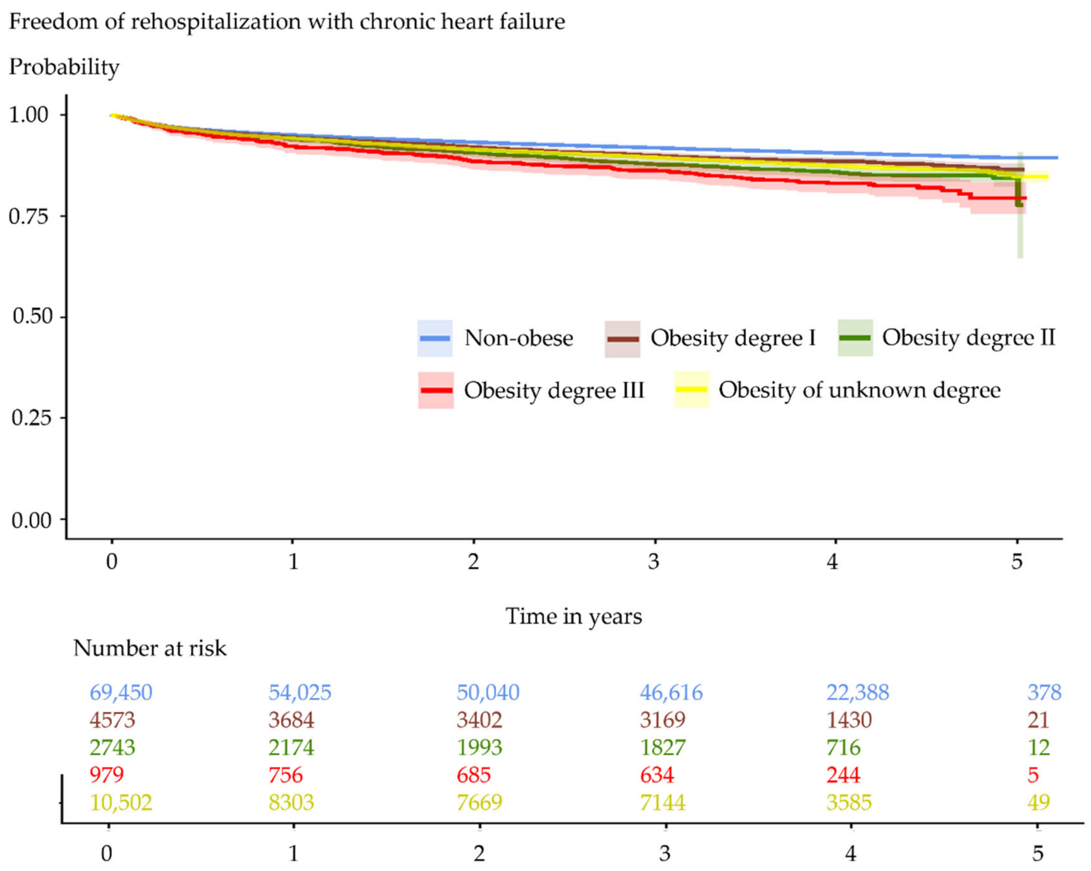

(c)

Figure S1: The overall survival rates (a), the estimated cumulative incidence of the primary outcome 'freedom from chronic heart failure (CHF)' (b) and the estimated cumulative incidence of the primary outcome 'freedom from rehospitalization due to chronic heart failure (CHF)' (c) stratified between patients with obesity degree I (BMI ≥ 30 to < 35 kg/m²; depicted in brown), obesity degree II (BMI ≥ 35 to < 40 kg/m², depicted in green), obesity degree III (BMI ≥ 40, depicted in red), obesity of unknown degree (depicted in yellow), and without obesity (depicted in blue). These estimates were derived using competing risk models where death was considered as a competing risk.
