# Supplementary material for: BMI-Stratified Exploration of the ‘Obesity Paradox’: Heart Failure Perspectives from a Large German Insurance Database
Source: J Clin Med. 2024 Apr 3;13(7):2086. doi: 10.3390/jcm13072086 (PMC11012389; doi:10.3390/jcm13072086)
Supplement: Supplementary file 1 [file jcm-13-02086-s001.zip › Supplement_TablesS1-5_Revision.pdf]

Table S1. Used Health Problems 10th Revision (ICD-10 GM) codes.

Health Problems 10th Revision (ICD-10 GM)	Code
Arterial hypertension (AHT)	I10.-, I11.-, I12.-, I13.-, I15.-
Diabetes mellitus (DM)	E10.-, E11.-, E12.-, E13.-, E14.-
Dyslipidemia	E78.-
Nicotine abuses	F17.-
Myocardial Infarction (MI)	I21.-, I22.-
Previous stroke	I63., I64.-, I69.3, I69.4
Chronic Coronary syndrome (CCS)	I25.-
Atrial flutter/fibrillation (AFL/AF)	I48.-
Peripheral artery disease (PAD)	I70.2-
Cerebrovascular disease (CeVD)	I65.-, I66.-, I67.2,
Chronic kidney disease (CKD)	N18.-, N19.-
Acute renal failure (ARF)	N17
Ischemic cardiomyopathy (ICM)	I25.5
Dilated cardiomyopathy (DCM)	I42.0
Hypertrophic obstructive cardiomyopathy (HOCM)	I42.1
Hypertrophic cardiomyopathy (HCM)	I42.2
Arrhythmogenic right ventricular cardiomyopathy (ARVC)	I42.80
Obesity	E66.-
Obesity grade I	E66.00
Obesity grade II	E66.01
Obesity grade III	E66.06, E66.07, E66.08
Obesity unspecified	E66.09
Iron deficiency	D50.0
Obstructive sleep apnea syndrome (OSAS)	G47.31
Left bundle branch block (LBBB)	I44.7
Left/right heart failure (L/RHF)	I50.1/I50.0
Malignancies	C00-C97

Presence of electronic cardiac device	Z95.0
Dyspnea	R06.0
Oedema	R60.-+R18
NYHA classes I/II/III/IV	I50.11/ I50.12/ I50.13/ I50.14
Shock	R57.0

Table S2. Used Anatomical Therapeutic Chemical classification system (ATC) codes.

Anatomical Therapeutic Chemical classification system (ATC)	Code
Platelet activation inhibition (PAI)	B01AC
Oral anticoagulation (OAC)	B01AE, B01AA, B01AF
Angiotensin receptor inhibitors/ angiotensin receptor blocker (ACEi/ARB)	C09
Betablockers	C07
Diuretics	C03A, C03B, C03E
Ivabradine	C01EB17
Mineralocorticoid receptor antagonist (MRA)	C03DA
Sodium glucose cotransporter-2 inhibitor (SGLT-2i)	A10BK
Digitalis	C01AA
Statin	ATC C10AA

Table S3. Used German procedure classification system (OPS) codes.

German procedure classification system (OPS)	Code
Percutaneous coronary intervention (PCI)	8-83
Coronary artery bypass grafting (CABG)	5-36
Renal replacement therapy	8-853, 8-855, 8-854
Left ventricle support	8-83
Resuscitation	8-77

Table S4. Hazard ratios of the different obesity stages regarding Cox regression models for freedom from rehospitalization.

New York Heart Association (NYHA)	Obesity degree I	Obesity degree II	Obesity degree III	Obesity of unknown degree
No NYHA	1.19 (1.04; 1.34)	1.45 (1.25; 1.69)	2.0 (1.59; 2.50)	1.29 (1.18; 1.41)
NYHA I	1.74 (0.99; 3.06)	1.45 (1.25; 1.69)	2.0 (1.59; 2.50)	1.29 (1.18; 1.41)
NYHA II	0.90 (0.64; 1.27)	0.93 (0.37; 2.36)	2.70 (1.03; 7.09)	1.62 (1.10; 2.37)
NYHA III	0.98 (0.78; 1.22)	1.21 (0.79; 1.86)	1.29 (0.67; 2.48)	1.06 (0.83; 1.34)
NYHA IV	0.89 (0.73; 1.08)	1.30 (1.01; 1.68)	1.79 (1.29; 2.48)	1.17 (1.01; 1.34)

Table S5. Hazard ratios of the different obesity stages regarding Cox regression models for overall survival

New York Heart Association (NYHA)	Obesity degree I	Obesity degree II	Obesity degree III	Obesity of unknown degree
No NYHA	0.86 (0.79; 0.93)	0.95 (0.86; 1.26)	1.06 (0.89; 1.26)	0.97 (0.92;1.03)
NYHA I	0.73 (0.43; 1.24)	1.11 (0.63; 1.96)	1.17 (0.51; 2.70)	1.00 (0.72; 1.37)
NYHA II	0.80 (0.61; 1.04)	1.08 (0.77; 1.52)	1.32 (0.76; 2.30)	0.78 (0.64; 0.94)
NYHA III	0.99 (0.84; 1.16)	0.96 (0.77; 1.19)	1.28 (0.95; 1.73)	0.89 (0.79; 1.00)
NYHA IV	0.90 (0.80; 1.02)	0.99 (0.86; 1.13)	0.98 (0.78; 1.24)	0.98 (0.91;1.06)
